# The Effect of Whitlockite as an Osteoconductive Synthetic Bone Substitute Material in Animal Bony Defect Model

**DOI:** 10.3390/ma15051921

**Published:** 2022-03-04

**Authors:** Jeong-Kui Ku, Il-hyung Kim, Jung Hee Shim, Yu ha Kim, Baek Hyun Kim, Young-Kyun Kim, Pil-Young Yun

**Affiliations:** 1 Department of Oral and Maxillofacial Surgery, Gangnam Severance Hospital, Yonsei University Health System, Seoul 06273, Korea; kujk123@gmail.com; 2 Department of Oral and Maxillofacial Surgery, Armed Forces Capital Hospital, Armed Forces Medical Command, Seongnam-si 13574, Korea; haonflower@gmail.com; 3 R&D Center, OSFIRM Co., Ltd., Seongnam-si 13605, Korea; jhshim@osfirm.co.kr (J.H.S.); yhkim@osfirm.co.kr (Y.h.K.); bhkim82@cgbio.co.kr (B.H.K.); 4 Department of Oral and Maxillofacial Surgery, Section of Dentistry, Seoul National University Bundang Hospital, Seongnam-si 13620, Korea; kyk0505@snubh.org; 5 Department of Dentistry and Dental Research Institute, School of Dentistry, Seoul National University, 101 Daehak-ro, Jongno-gu, Seoul 03080, Korea

**Keywords:** biphasic calcium phosphate, beta-tricalcium phosphate, hydroxyapatite, whitlockite

## Abstract

This study aimed to evaluate the biomechanical properties in vitro and the bone regeneration of whitlockite (WH) compared with hydroxyapatite (HA) or β-tricalcium phosphate (β-TCP)-based material. We investigated the morphology and phase composition of the bone grafts using a scanning electron microscope and X-ray diffractometer patterns and tested the compressive strength. Four circular defects of 8 mm in diameter were created on the calvaria of twelve rabbits. One defect was left empty, and each of the other defects was filled with WH, HA, and β-TCP. At 4 and 8 weeks, the specimens were harvested to evaluate for the new bone formation and the remaining bone grafts. Regarding the biomechanical properties, the three grafts had a similar micropore size, and WH showed nanopores. The compressive strength of WH was higher than HA and β-TCP without statistical significance. The radiological and histomorphometric analyses demonstrated that the new bone formation was similar among the groups. The remaining bone graft of the WH group was greater than that of the HA and β-TCP groups at 4 weeks (*p* < 0.05), and the total bone area of the WH, HA, and β-TCP groups was greater than that of the other (*p* < 0.01). WH has excellent volumetric stability and osteoconductivity compared with HA and β-TCP.

## 1. Introduction

Autologous bone, which has osteogenesis, osteoinduction, and osteoconduction abilities, is still considered the gold standard bone graft treatment [1,2,3]. Bone tissue engineering has been researched for developing bone substitutes to overcome several disadvantages of autografts, such as extended healing time and the need for multiple surgeries.

Alloplastic bone substitutes, emulating the physicochemical properties of bone tissues, have been widely used because of their attractive advantages such as few donor site morbidity, little risk of transmission of infectious diseases, and sufficient supply [4]. The optimal bone substitutes possess biocompatibility with volumetric stability and allow cell infiltration for the remodeling process. The alloplastic bone substitutes have various osteoconductive capabilities depending on the manufacturing methods, crystal structure, size of pores, mechanical properties, composition, and absorption rate [5]. Among them, synthetic calcium phosphate ceramics (hydroxyapatite (HA) and β-tricalcium phosphate (β-TCP)) have been widely used in implant dentistry because they have a similar composition to inorganic component of the tissue and excellent biocompatibility [6].

Whitlockite (WH) is a calcium orthophosphate crystal, Ca_9_Mg(HPO_4_)(PO_4_)_6_, with an unusual form of calcium phosphate with an unknown biological role. Although WH is a relatively rare mineral in nature, it is the second most abundant mineral in human bone (approximately 20–35% of the total weight) followed by HA, and it is particularly found in bone with elevated dynamic loading [5]. In 2017, the synthetic WH was shown to recapitulate the early stage of bone regeneration by stimulating osteogenic differentiation, prohibiting osteoclastic activity, and transforming into mechanically enhanced HA-neo bone tissue under physiological conditions [7]. In the rat model, the WH-reinforced composite scaffold showed comparable bone regeneration with intermediate resorbability compared with HA and β-TCP [8].

The authors hypothesized that the synthetic WH could act as an osteoconductive bone graft substitute with high mechanical stability and bioactivity compared with synthetic calcium phosphate ceramics. This study aimed to evaluate the biomechanical characteristics and bone healing outcomes of WH compared with HA and β-TCP in the rabbit calvarial defect model.

## 2. Materials and Methods

Guidelines regarding the care of animal research subjects were strictly followed and approved by the Institutional Animal Care and Use Committee of Seoul National University Bundang Hospital, Seongnam-si, Korea (IACUC No. BA1901-264/004-01).

### 2.1. Fabrication of WH Graft

MagOss (100% WH, OSFIRM Co., Ltd., Seongnam, Korea) was prepared by sintering the polyurethane sponges infiltrated with the WH slurry containing vehicle, binder, and dispersant. Specifically, the vehicle and binder were mixed with a dispersant to form the first slurry. The first slurry prepared was deagglomerated by stirring and subsequently deaired until no further release of air bubbles. The WH powder was added to the first slurry to form the second slurry with the following ratio: WH powder: first slurry = 1:1.2. The second slurry was mixed by planetary mixer and three-roll mill until completely mixed. Polyurethane foams that were cut into a desired shape and size were immersed in the second slurry to the first coat. The coated polyurethane foams were completely dried and first sintered at 750 °C for 2 h to eliminate the matrix. Then, the sample was second coated by dipping and was completely dried. After drying, the samples were second sintered at 750 °C for 2 h to increase the compressive strength and crystallinity of porous bodies. Finally, the sintered bodies were crushed to 0.6–1.0 mm and sterilized by gamma irradiation.

### 2.2. Characterization of Bone Graft

#### 2.2.1. Scanning Electron Microscope (SEM)

Bone graft morphology was investigated by SEM, using a Philips XL-30-FEG SEM (Philips, Hillsboro, OR, USA) at an accelerating voltage of 15 kV.

#### 2.2.2. X-ray Diffractometer (XRD)

The phase composition of the grafts was determined using an XRD (Smart Lab, Rigaku, Neu-Isenburg, Germany). XRD patterns were recorded at room temperature using Cu Kα1 radiation with the following measurement conditions: a tube voltage of 40 kV, a tube current of 40 mA, a step size of 0.02, and a scan speed of 3°/min over an angular range of 20–80°.

#### 2.2.3. Compressive Strength

The compressive strength of grafts was measured via a universal testing machine (Test One UTM, Test One, Siheung, Korea) using cuboid specimen (40 × 60 × 5 mm^3^) with the following measurement conditions: loaded area on this sample of 400 mm^2^ (WH and HA) or 249 mm^2^ (β-TCP), speed of the load of 0.5 mm/min, and distance limitation of 2.5 mm.

### 2.3. Animal Preparations and Experimental Design

Studies supported the fact that rabbits show promise as an animal model for the comparison between three or more bone implant materials because four bony defects were formed at least. Hence, rabbits were selected as animal models. In the study, 12 New Zealand white conventional rabbits (5–6 months old, weighing 3.0–3.5 kg in good health) were used. Rabbits were fed with a commercial diet (Rabbit Chow GoldPet, #35520, Cargill Agri Purina, Inc., Pyungtaek, Korea) and housed in individual cages.

The animals were prepared with a subcutaneous injection of atropine 0.005 mg/kg (Daihan Pharm Co., Ansan, Korea) under a supine position and anesthetized with an intramuscular injection of alfaxalone (Alfaxan, Virbac, Carros, France) 5.0 mg/kg and xylazine (Rompun, Bayer Korea, Ansan, Korea) 5.0 mg/kg after 15 min. After endotracheal intubation of a 5.0-sized tube, general anesthesia was maintained with sevoflurane 2.2% (JW Pharmaceutical, Hwasung, Korea) and oxygen level at 2.0 L/min. Before surgical procedures, the animals were injected intramuscularly with cefazolin 30 mg/kg (Chongkundang Pharm, Cheonan, Korea).

**Figure 1:**
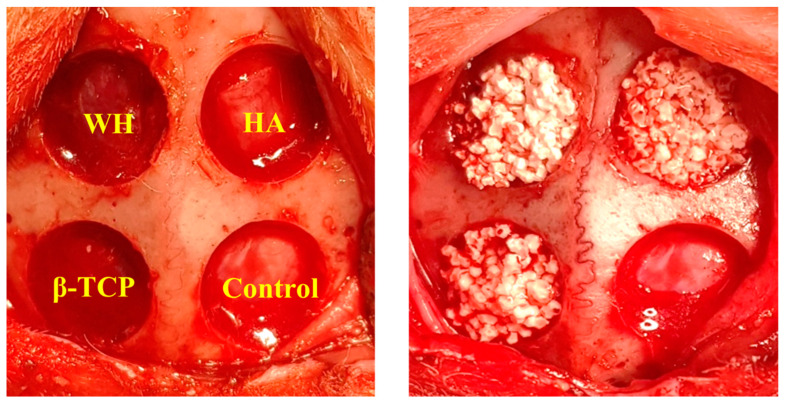
Intraoperative images. Four circular defects of 8 mm in diameter were created. Each defect was filled with whitlockite (WH), hydroxyapatite (HA), and β-tricalcium phosphate (β-TCP). Sham surgery control defect was filled with blood clots.

The surgical field was scrubbed with povidone–iodine solution. Local anesthesia for hemostasis using 2% lidocaine with 1:100,000 epinephrine (Yuhan Co., Ltd., Seoul, Korea) was injected into both calvarial areas. Skin incisions were made on the calvaria, and subperiosteal dissection was raised to expose the calvarial surface. Four holes were prepared using an 8.0-mm-diameter trephine bur (Dentium, Suwon, Korea) with profuse saline irrigation. Each defect depth proceeded until the inner cortex is completely removed. Cold saline irrigation was performed during burring to minimized heat generation (Figure 1).

One bony defect was left empty as a negative control, and each of the other defects was filled with the following three synthetic bone materials, which are high purity bone substitutes with a similar particle size (0.6–1.0 mm):

(1) WH (Ca_9_Mg(HPO_4_)(PO_4_)_6_): MagOss (OSFIRM Co., Ltd., Seongnam, Korea) with 58.6% porosity, comprising nanoporous (100–1000 nm) and microporous (100–1000 μm) structure.
(2) HA (Ca_10_(PO_4_)_6_(OH)_2_): Bongros-HA (CGBio, Seongnam, Korea) with 69.9% porosity and 300 μm of the porous structure.
(3) β-TCP (Ca_3_(PO_4_)_2_): Excelos (CGBio, Seongnam, Korea) with 56.8% porosity and 100–300 μm of the porous structure.

Primary closure was performed using polyglactin 4-0 (Vicryl, Ethicon, Menlo Park, CA, USA) for all surgical sites. Postoperatively, 1.0 mL of dexamethasone-21-isonicotinate (Voren, Boehringer Ingelheim Korea Ltd., Seoul, Korea) was injected once, and clemizole penicillin G and sodium penicillin G (Antipen-SM, WooGene B&G Ltd., Seoul, Korea) 0.1 mL/kg were injected three times on every third day. Furthermore, six rabbits were sacrificed each time on 4 and 8 weeks later through formalin perfusion to evaluate bone remodeling. Euthanasia was performed via air injection into the ear vein with a 50 mL syringe.

### 2.4. Microcomputed Tomography Evaluation

Microcomputed tomography (micro-CT) was taken immediately with a 16-slice multidetector CT scanner (SkyScan, Kontich, Belgium; 130 kV, 60 mA, 500 ms, and 0.75 mm thickness) after harvesting block sections (8.0 × 8.0 × 5.0 mm^3^), including the grafted sites. Fixed specimens were scanned with an aluminum filter (1.0 mm) for high resolution.

**Figure 2:**
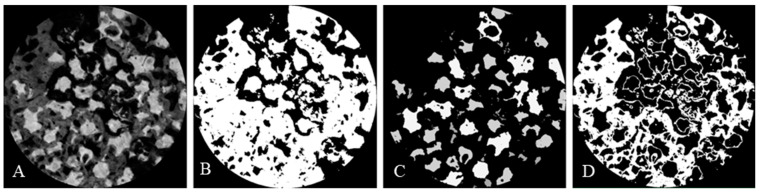
Micro-CT analysis process. (**A**) Reconstructed original image. (**B**) Image adjusted to show both bone substitutes and newly formed bone. (**C**) Image adjusted to show only bone substitutes. (**D**) Image adjusted to show only newly formed bone.

Referring to the reconstructed CT image, only the experimental area of 8 mm diameter was reconstructed into the 3D model. The image analysis was performed using SPOT Software V4.0 (Diagnostic Instruments Inc., Sterling Heights, MI, USA). The volume of the isolated new bone was measured as CAD data using Image-Pro Plus Version 7.0 (Media Cybernetics Inc., Rockville, MD, USA) analysis software. The measurements included the following parameters of area ratio per total cavity volume of interest (ø 8 mm): (i) newly formed bone volume (NV); (ii) remaining bone substitute volume (BV); and (iii) total bone volume (TV), calculated from the sum of NV and BV (Figure 2).

### 2.5. Histological Analysis

The specimens were fixed with 10% buffered neutral formalin (Sigma Aldrich Co. LLC., St. Louis, MO, USA) for 1 week. The specimens were decalcified in formic acid (Shandon TBD-1, Thermo Fisher Scientific Inc., Kalamazoo, MI, USA) following water rinse and in tissue processor (Shandon Citadel 2000, Thermo Fisher Scientific Inc., Kalamazoo, MI, USA) and embedded in paraffin using embedding center (Shandon Histocentre 3, Thermo Fisher Scientific Inc., Kalamazoo, MI, USA). Serial sections of 3.0 µm thickness were cut using a microtome (Shandon Finesse 325, Thermo Fisher Scientific Inc., Kalamazoo, MI, USA), and each sample was stained with hematoxylin and eosin. The samples were scanned using a digital slide scanner (3DHISTECH Ltd., Budapest, Hungary) and observed using CaseViewer (3DHISTECH Ltd., Budapest, Hungary) with magnification (×3.5). Tissue sample images were taken for histological examination (×12.5) and histomorphometric measurement (×40).

### 2.6. Histomorphometric Measurement

Images of mid-section the specimens of each sample were acquired from a light microscope (BX51, Olympus Co., Tokyo, Japan) connected to a computer, CCD camera (SPOT Insight 2 Mp scientific CCD digital camera system, Diagnostic Instruments Inc., Sterling Heights, MI, USA), and adaptor (U-CMA3, Olympus Co., Tokyo, Japan) to obtain histomorphometric measurements. The measurements were conducted by blinded trained examiner (I.-h. Kim) s to the group allocation. Similar to the CT analysis, the measurements included the following parameters of area ratio: (i) NV and (ii) TV (the sum of NV and remaining BV).

### 2.7. Statistical Analysis

Statistical analyses were performed on the micro-CT and histomorphometric results by the Kruskal–Wallis test using the Statistical Package for the Social Sciences (SPSS) version 25.0 software (SPSS, Inc., Chicago, IL, USA), and a value of *p* = 0.05 was considered significant.

## 3. Results

### 3.1. Morphology and Composition Analysis

The bone graft products’ morphology was investigated using SEM. The SEM observations confirmed the results of the morphology of granules and macropores at lower magnification. The porous granules with a homogeneous size of 0.6–1.0 mm and the macropore more than 200 µm were observed in all of the bone graft products.

**Figure 3:**
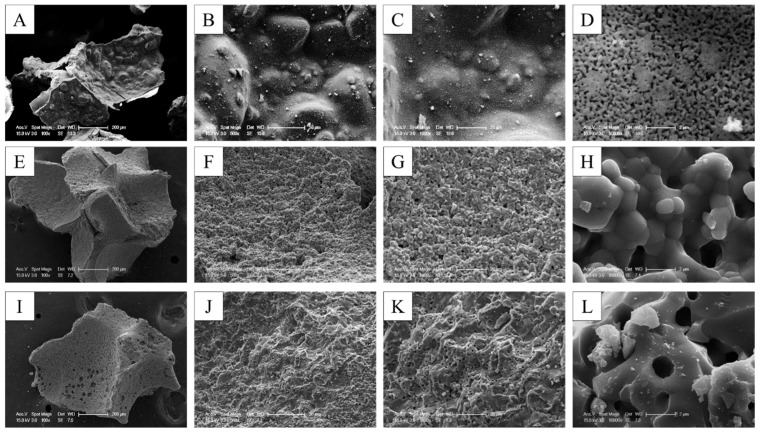
SEM images of different bone grafts. (**A**–**D**) 100% WH. (**E**–**H**) 100% HA. (**I**–**L**) 100% β-TCP.

The higher resolving power of SEM revealed additional details. With the SEM, it was possible to confirm not only the microstructure but also the nanostructure. WH showed a more homogeneous and smaller grain size when compared with the other two alloplastic products. The grain size of WH was confirmed to be approximately 0.3 µm, but the grain size of HA and β-TCP was confirmed to be 0.5–2 µm. The micropore of 10–200 µm was shown in three types of alloplastic products; however, the nanopore less than 0.5 µm was found in only WH because of the different sizes of the grain (Figure 3).

**Figure 4:**
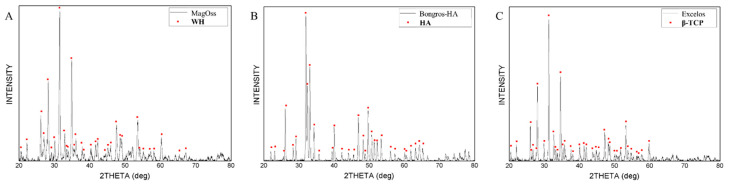
XRD patterns of different bone grafts. (**A**) 100% WH. (**B**) 100% HA. (**C**) 100% β-TCP.

The bone graft products’ composition analysis was investigated via XRD. The result of X-ray diffraction analysis appeared in different patterns in different alloplastic products. The crystalline structures of WH showed the WH pattern, the crystalline structure of HA showed the HA pattern, and the crystalline structure of β-TCP showed the β-TCP pattern. The secondary phase was not found. The peak for WH displayed a pattern that was more horizontally spread out. The peak for HA and β-TCP was sharper than that of WH, showing narrow width patterns because of each grain size (Figure 4).

### 3.2. Compressive Strength

**Figure 5:**
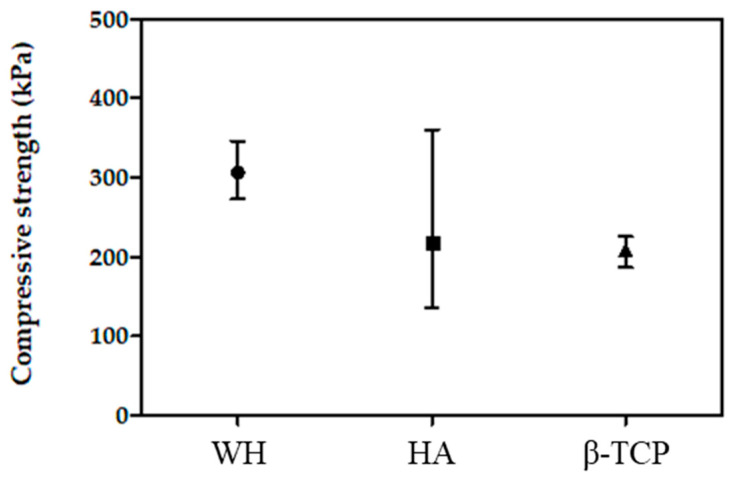
Compressive strength of different bone grafts.

The compressive strength values of WH were higher than those of other bone graft products. The compressive strength values of the WH were approximately 306.25 ± 39.3 kPa, those of the HA and β-TCP were approximately 216.63 ± 143.63 kPa and 208.03 ± 21.68 kPa (Figure 5). The mean compressive strength of WH were higher value than TCP and HA, although the statistical significance was lacking because of the high porosity of the specimens.

### 3.3. In Vivo Results 

All experimental animals showed no remarkable complications, such as infection, or hematoma, during the observation period. At 4 weeks, graft particles embedded in newly formed fibrous tissue on the bone defects grafted with WH, HA, and β-TCP were observed, whereas the left empty (negative control) site revealed that the bone defects were bridged circularly by newly formed hard tissue. At 8 weeks, closure of the defects with new bone was observed macroscopically at all graft sites. The newly formed hard tissue was macroscopically homogeneous without clear clinical distinction between the host bone boundary and residual graft particles, whereas the negative control showed incomplete bony healing.

#### 3.3.1. Micro-CT Measurement 

**Figure 6:**
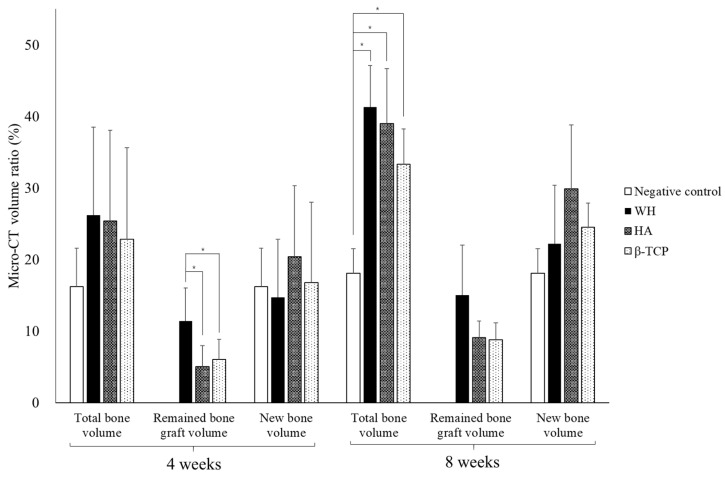
Micro-CT analysis of bone volume ratio according to the group. * *p* < 0.05.

At 4 weeks, no significant differences were observed between parameters that expressed NV and TV ratio, among the three experimental groups (Figure 6). However, the remaining BV was higher in WH (11.4 ± 4.6%) than that in HA and β-TCP (5.0 ± 3.0% and 6.0 ± 2.8%, respectively) (Table 1, *p* = 0.042).

At 8 weeks, the three experimental groups (41.3 ± 5.8%, 39.0 ± 7.7%, and 33.3 ± 5.0% on WH, HA, and β-TCP, respectively) showed a higher TV ratio than the negative control group (18.1 ± 3.4%) (Table 1, *p* = 0.005). BV was slightly higher on WH (15.0 ± 7.0%) and HA and β-TCP (9.1% ± 2.3% and 8.8 ± 2.4%, respectively). NV showed a similar aspect to that of 4 weeks.

#### 3.3.2. Histologic Analysis 

**Figure 7:**
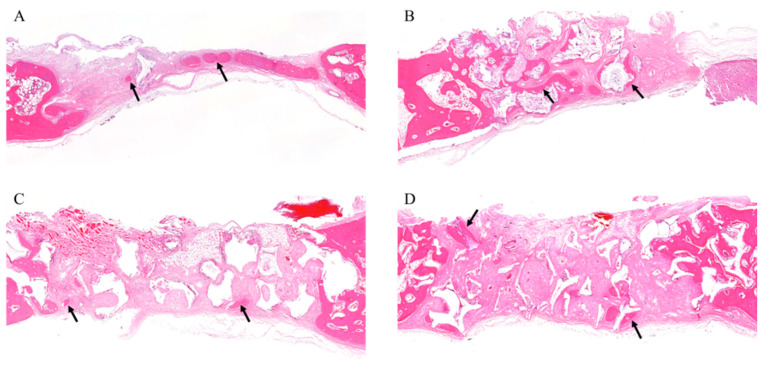
Histological analysis at 4 weeks. (**A**) Histological section of defects filled without graft materials. A small amount of immature new bone was formed (arrows). (**B**) Histological section of defects filled with WH. The regenerated bones surround the graft materials (arrows). (**C**) Histological section of defects filled with β-TCP. New bone formation was seen around the margin of the defect and graft material (arrows). (**D**) Histological section of defects filled with HA. New bone formation around the remaining graft material was seen in multiple sites (arrows).

In all samples, there was no significant inflammatory response, necrosis, or foreign body reactions. At 4 weeks, the grafts (WH, HA, and β-TCP) showed similar aspects; that is, the osteoconductive bone formation was observed surrounding the graft particles and fibrous connective tissues were shown to be dominant. The negative control showed that most of the defects were filled with loose connective tissue. A small amount of immature new bone was formed with inflammatory cell infiltration and vascular proliferation (Figure 7).

**Figure 8:**
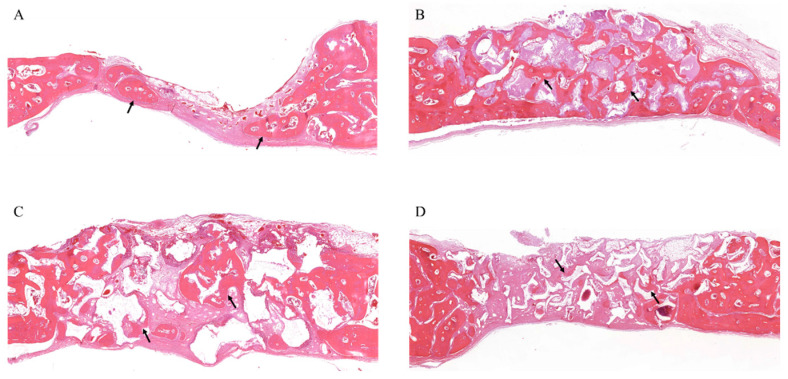
Histology at 8 weeks. (**A**) Histological section of defects filled without graft materials. New bone formation increased around the margin and adjacent regions, and the bone structure became more mature (arrows). (**B**) Histological section of defects filled with WH. The newly formed bone has a dense bony trabecula structure (arrows). (**C**) Histological section of defects filled with β-TCP. The amount of newly formed bone was increased, and it has a loose bony trabecula structure (arrows). (**D**) Histological section of defects filled with HA. Bone formation was increased around the graft material over the entire defect, and a loose bony trabecula structure was observed (arrows).

At 8 weeks, WH was resorbed and replaced with new bone, which was entirely covered with continuous bone tissue (Figure 8). The negative control showed a much mature bony structure, but the bony tissue was not regenerated up to the center of the defect (Figure 8A). The new bone around the remaining WH formed dense bony trabeculae (Figure 8B). Most β-TCP particles were resorbed, and the new bone showed loose bony trabeculae with an increasing aspect compared with that of 4 weeks (Figure 8C). The HA group showed that the new bone formation was observed throughout the defect around the remaining HA without resorption, and the new bone showed loose bony trabeculae (Figure 8D).

#### 3.3.3. Histomorphometric Measurement 

**Figure 9:**
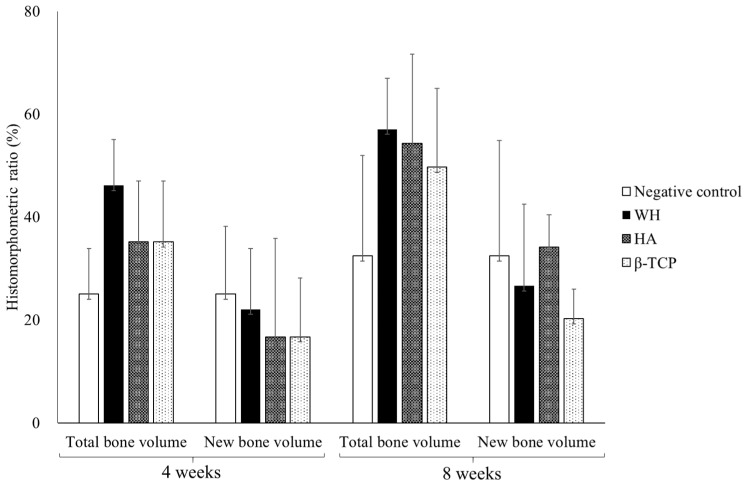
Histomorphometric measurement of bone volume ratio (%) according to the group.

All grafted experimental groups have higher TV without significance and similar NV compared with the negative control during 4 and 8 weeks (Figure 9). Although no statistical difference was observed among the experimental group, TV was higher in order of WH, HA, and β-TCP. Regarding NV, WH was higher than HA and β-TCP at 4 weeks, but HA was higher than the others at 8 weeks.

## 4. Discussion

Several synthetic bone substitutes have been suggested to enhance their mechanical stability, bone forming capacity, and osteoconductivity. However, optimal bone substitutes that could allow slow resorption with cell infiltration for neovascularization and new bone formation have not been proposed. The authors hypothesized that the mechanical and biological outcomes of WH were not inferior to those of synthetic calcium phosphate ceramics (HA and β-TCP), which are widely used in implant dentistry.

Porosity is an important factor that affects cell penetration, proliferation, and differentiation. Micropores, ranging from 20 to 1500 µm, have been used in bone tissue engineering development [9]. Although the diameter of osteoblasts is known as 10–50 µm, osteoblasts prefer the pore size on the order of 100–200 µm, which allows regenerating mineralized bone after grafts [10]. Similar to HA and β-TCP, WH showed an average pore size of 10–200 µm, which is within the optimal range for vascularization and bone ingrowth. Considering that small pores can stimulate greater ion exchange, bone protein adsorption, formation of nonmineralized osteoid or fibrous cell aggregation [10,11], the nanopores of WH (<0.5 µm) could provide more advantages compared with those without nanopores.

To characterize the mechanical properties, the compressive strength is the most often analyzed property for bone substitutes. Generally, the compressive strength ranges between 90 and 230 MPa and 2 and 45 MPa in human cortical and cancellous bones, respectively [12]. Calcium phosphates are known as limited biomechanical support because they have little tensile strength. In this study, the compressive strength of the WH (0.306 ± 0.039 MPa) was slightly higher than the HA and β-TCP (0.216 ± 0.144 and 0.208 ± 0.017 MPa, respectively). Although the method of measuring the compressive strength has a difference in the size of the load area (WH, HA: 400 mm^2^, β-TCP: 249 mm^2^), it was considered that β-TCP would show lower compressive strength even if it was measured under a wider load area condition of WH or HA. Thus, WH could provide a more stable and favorable condition to maintain space during bone regeneration compared with other alloplastic bone substitutes.

Synthetic bone substitutes have only osteoconductive properties, which indicate mechanical stability with biocompatibility for the migration of osteogenetic cells and an advantage for space maintainability [13,14,15]. HA accounts for 65% of the bone matrix and remains for a long time with slow absorption. The larger the crystallinity, the longer the absorption period [16]. However, the slow absorption could interrupt the new bone formation and remodeling processes. However, TCP, a composition of calcium and phosphorus ions in a ratio of 3 and 2, has various absorption periods of 3–24 months depending on the chemical structure, porosity, and particle size of the material [16]. Some products were mixed with HA and TCP with a ratio varying from 2:8 to 7:3 to overcome their disadvantages [5]. In the mixed products, HA acts as mechanical support until maintaining the structural stability of the new bone tissue, and TCP could increase the adhesion surface of an osteoblast through rapid resorption [5,16]. For predictable clinical outcomes, the composition and quality of the materials should be constant, but it is difficult to strictly control the composition of the mixed products [16]. To overcome these problems, various investigations attempted to change the synthetic bone as block, cement, pastes, powder, granules, and putty type with carboxymethyl cellulose or hyaluronic acid [6].

Recent efforts have been performed to easily synthesize WH, which has been difficult to fabricate and research in low-temperature conditions [8]. WH is a calcium phosphate-based ceramic that contains a magnesium ion and has higher compressive strength than HA [8,17]. Because magnesium ions play a role in decreasing the activity of osteoclasts [18], WH was expected to affect bone substitutes. Kim et al. [7] reported that WH nanoparticles could stimulate osteogenic differentiation, prohibit the osteoclastic activity, transform into mechanically enhanced neo bone tissues, and more enhance osteogenic activity compared with HA. In accordance with previous research, our study demonstrated that WH has great initial stability compared with HA and β-TCP. In terms of TV, WH showed effective osteoconductivity, not inferior to conventional synthetic calcium phosphate ceramics.

In 2018, HA and WH nanoparticles with hydrogel scaffolds were reported to significantly enhance cellular growth and osteogenic differentiation [19]. Although the bone healing ability of WH alone was comparable with HA, various applications should be applied to enhance osteoconductivity through the change of fabrication method, porosity, crystal structure, and mixing with other scaffolds or growth factors. As 3-dimentional printing technology has widely applied on the various composite bone scaffolds and several including sinusoidal magnetic field [20,21], clinical application of 3D printing alloplastic bone substitutes has been proposed [22]. Therefore, the 3D technology could be developed by using WH with its proven osteoconductivity, to wide-range clinical application for implant dentistry. Within the limitations of this preclinical study, we confirmed the effectiveness of WH in terms of bone formation capacity and volumetric stability. Further clinical studies are needed to compare with other bone grafts based on our results.

## 5. Conclusions

The biomechanical property and osteoconductive bone healing capacity of WH were not inferior to those of synthetic calcium phosphate ceramics. WH could be applied with volumetric stability to alter HA and β-TCP as osteoconductive alloplastic bone substitutes.

## Figures and Tables

**Table 1:** Micro-CT analysis of bone volume ratio (%) according to the group.

Group	4 Weeks			8 Weeks		
	TV	BV	NV	TV	BV	NV
Control	16.2 ± 5.4	–	16.2 ± 5.4	18.1 ± 3.4	–	18.1 ± 3.4
WH	26.2 ± 12.3	11.4 ± 4.6	14.7 ± 8.1	41.3 ± 5.8 *	15.0 ± 7.0	22.2 ± 8.2
HA	25.4 ± 12.7	5.0 ± 3.0 ^†^	20.4 ± 9.9	39.0 ± 7.7 *	9.1 ± 2.3	29.9 ± 8.9
β-TCP	22.8 ± 12.8	6.0 ± 2.8 ^†^	16.8 ± 11.2	33.3 ± 5.0 *	8.8 ± 2.4	24.5 ± 3.4
*p*	0.389	0.042	0.699	0.005	0.403	0.059

TV: total bone volume, BV: bone substitute volume, NV: newly formed bone volume. * Statistically significant differences compared with the control group by the Kruskal–Wallis test. ^†^ Statistically significant differences compared to the WH group by the Kruskal–Wallis test.

## Data Availability

Not applicable.
